# Pulmonary Alveolar Proteinosis due to Familial Myelodysplastic Syndrome with resolution after stem cell transplant

**DOI:** 10.4322/acr.2021.382

**Published:** 2022-05-13

**Authors:** Amjad Basheer, Eduardo Messias Hirano Padrao, Kangwook Huh, Susan Parker, Tejal Shah, Daniel A. Gerardi

**Affiliations:** 1 University of Connecticut, Department of Internal Medicine, Farmington, Connecticut, USA; 2 Hartford Healthcare, Department of Pathology, Hartford, Connecticut, USA; 3 Hartford Healthcare, Department of Pulmonology, Hartford, Connecticut, USA; 4 Saint Francis Hospital, Section of Pulmonary Critical Care Medicine, Hartford, Connecticut, USA

**Keywords:** Pulmonary alveolar proteinosis, Stem cell transplantation, GATA2 deficiency, Myelodysplastic Syndromes, Bronchoalveolar Lavaga

## Abstract

Pulmonary alveolar proteinosis (PAP) is a rare lung disease with an incidence of 0.2 cases per million. PAP has multiple causes, including autoimmune, hereditary, congenital, or secondary. The latter includes hematologic conditions and exposure to different kinds of dust. Most patients present fever, dyspnea, and cough. The chest computed tomography (CT) may reveal the crazy-paving polygonal shapes with superimposed ground glass opacities delimited by thickened interlobular septa; however, this finding is more prevalent in patients with autoimmune PAP. Bronchoalveolar lavage (BAL) shows a milky-opaque appearance with PAS-positive debris on cytology. Treatment is focused on the underlying disease; however, some patients may require whole lung lavage for symptomatic management. We report a case of a 30-year-old female with a history of familial myelodysplastic syndrome (MDS) with GATA 2 mutation who presented to the outpatient clinic with several months of progressive dyspnea and nonproductive cough. The chest CT revealed bilateral ground-glass opacities prominently in the upper lobes. She underwent a bronchoscopy with lavage and biopsy, which revealed fragments of lung parenchyma with intra-alveolar coarse granular eosinophilic material strongly positive for PAS and d-PAS. The overall clinical presentation and histologic findings were diagnostic of PAP. Her GM-CSF was negative, and due to her history of MDS, secondary PAP (S-PAP) was strongly suspected. She underwent a successful allogeneic bone marrow pluripotent stem cell transplant to treat the myelodysplastic syndrome, with a follow-up chest CT showing clear lung parenchyma. The patient had resolution of symptoms about four months after the bone marrow transplant, confirming the diagnosis of S-PAP.

## INTRODUCTION

Pulmonary alveolar proteinosis (PAP) is a rare lung disease characterized by dysfunction of alveolar macrophages resulting in intra-alveolar accumulation of surfactant. First described in 1958, this entity currently has an incidence of 0.2 cases per million.^1^ PAP can be classified into 3 groups: (a) disruption of Granulocyte-macrophage colony-stimulating factor (GM-CSF); (b) disorder of surfactant production; (c) secondary PAP (S-PAP).^2^ Secondary PAP include exposure to a different kind of dust such as silica, titanium, indium oxide, and some hematologic disorders.^3^ We report a case of S-PAP due to familial myelodysplastic syndrome (MDS) with GATA2 deficiency that resolved after hematopoietic stem cell transplantation.

## CASE REPORT

A 30-year-old woman presented to the emergency department with three days of fever and chills. She was evaluated at the pulmonology clinic for chronic dyspnea on exertion and a nonproductive cough. Her physical examination revealed an elevated body mass index (BMI) of 33 kg/m^2^, fever (39.1 °C), respiratory rate of 20 inspirations per minute, and tachycardia of 110 beats per minute. She was hemodynamically stable with a blood pressure of 142/88 mmHg. On respiratory examination, she had normal lung sounds and was comfortable in room air, saturating 94%. She had a known history of familial MDS with GATA 2 mutation and was a former smoker with a history of five pack-years.

**Figure 1:**
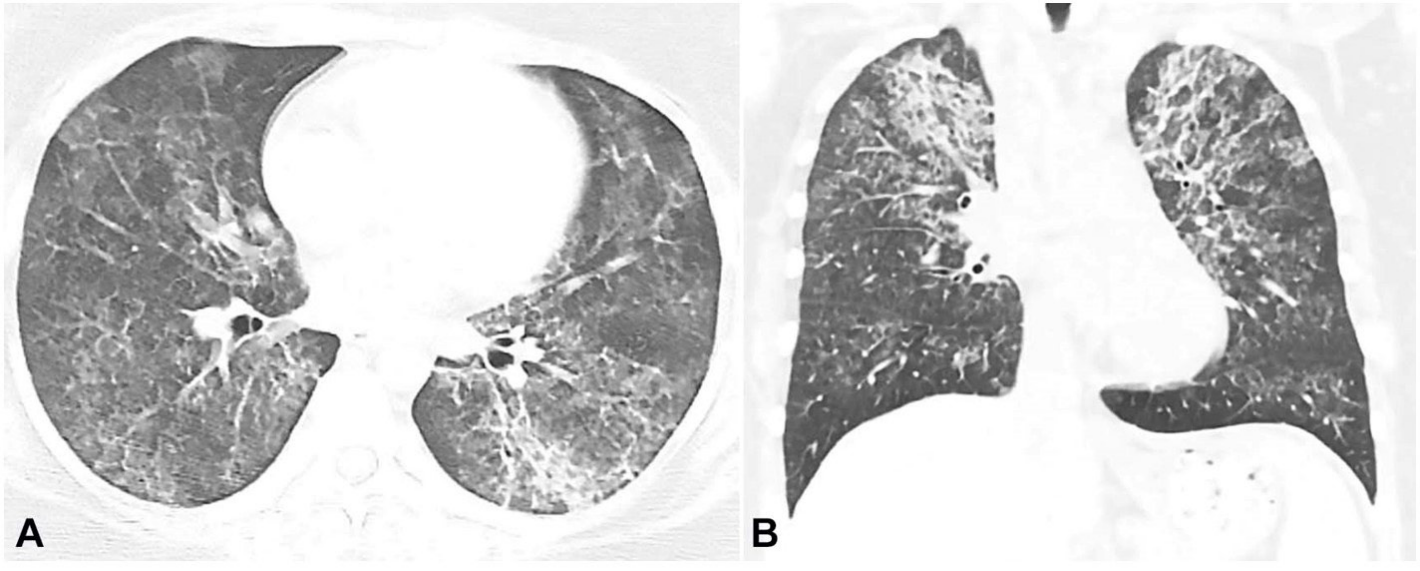
Chest CT. **A –** axial plane – **B –** coronal plane -showing bilateral diffuse mixed interstitial and alveolar opacities, predominantly in the upper lobe.

Her complete blood count revealed leukocytes of 2700/uL (RR 4000-11000/uL) with neutropenia. The chest CT showed bilateral alveolar and interstitial opacities involving both the upper and lower lobes (Figure 1), confirmed by a high-resolution computerized tomography.

**Figure 2:**
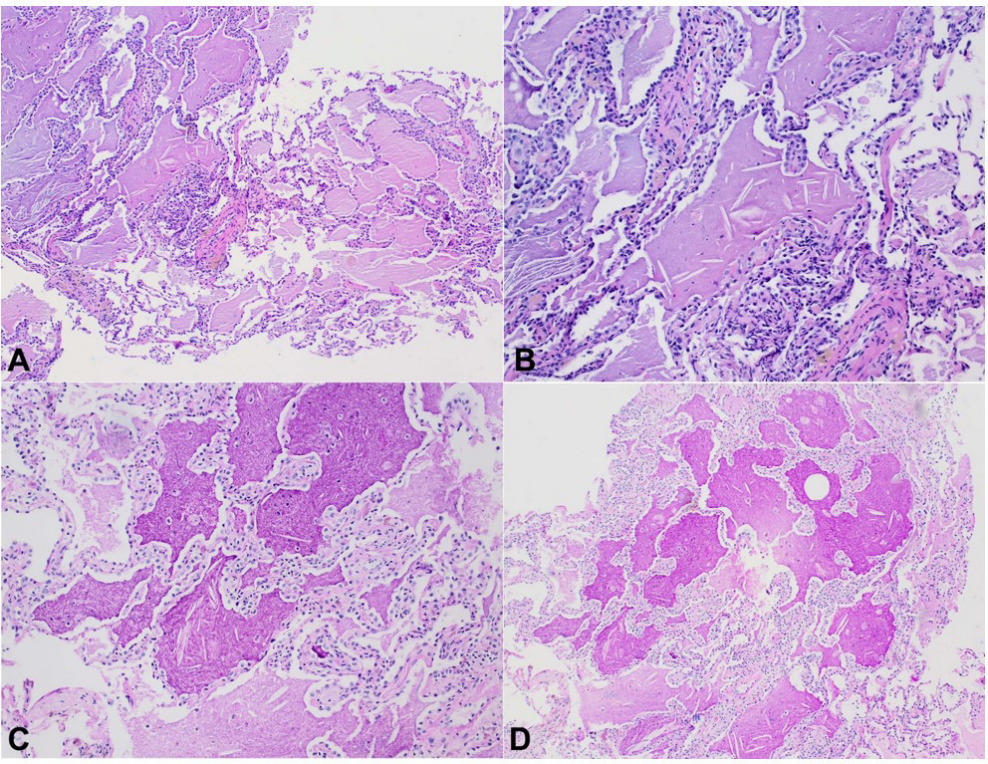
Photomicrographs of the lung. **A** (100x) and **B** (200x) - H&E stain shows alveoli completely filled with amorphous eosinophilic material. A few scattered macrophages, as well as cholesterol clefts, are present within the eosinophilic material. There is a minimal interstitial inflammatory cell infiltrate, there are no granulomas and no fibrosis. Note that the basic alveolar architecture is preserved. **C** (200x) and **D** (100x) - Fragments of Lung Parenchyma showing intra-alveolar filling with coarsely granular eosinophilic material that is strongly positive on PAS and d-PAS.

She underwent bronchoscopy with lavage and biopsy, which depicted lung parenchymal fragments with intra-alveolar coarse granular eosinophilic material strongly positive for PAS and d-PAS. No microorganisms were identified on other stains for acid-fast bacilli or for fungi (Figure 2). The anti-GM-CSF was negative. The overall clinical presentation and characteristic histologic findings were suggestive of S-PAP.

**Figure 3:**
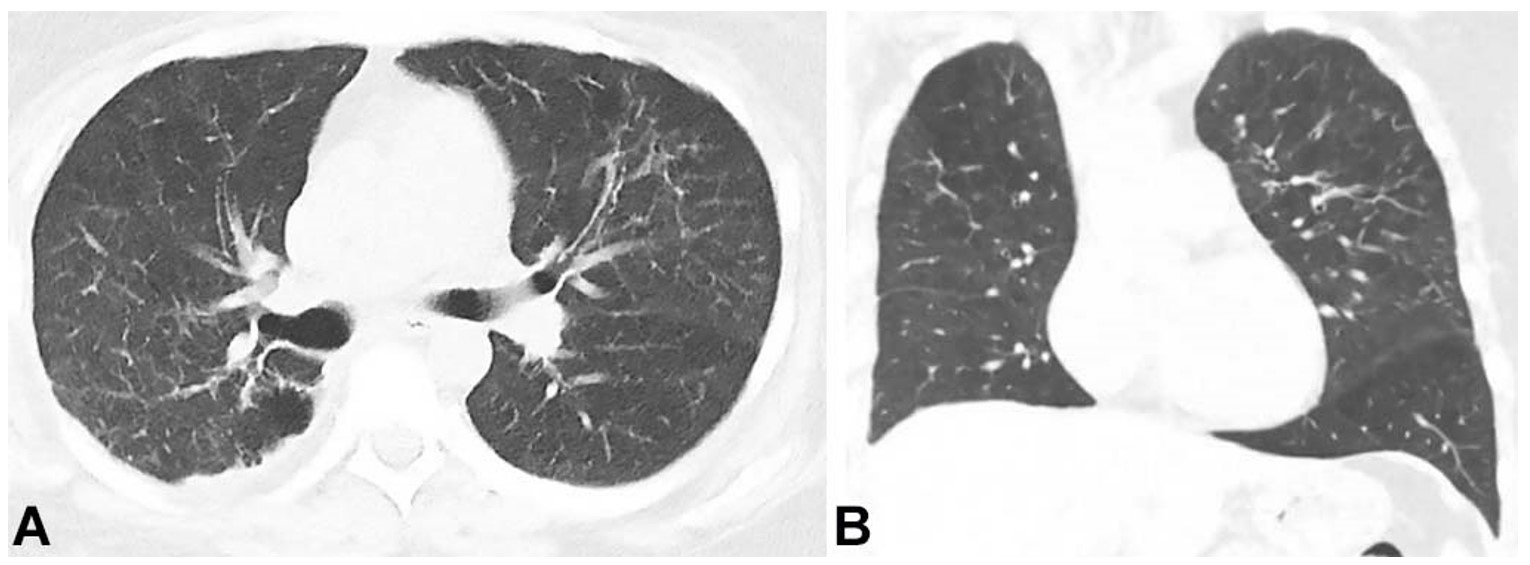
Chest CT. **A –** axial plane, **B –** Coronal plane; showing a marked decrease of the patchy ground-glass attenuation and interstitial thickening in both lungs​.

She underwent a whole lung lavage for symptom relief and, subsequently, a successful allogeneic bone marrow pluripotent stem cell transplantation as a therapeutic choice for the MDS. Due to the possible diagnosis of S-PAP, it was expected that transplantation would also improve her respiratory symptoms. Her follow-up chest CT revealed near-complete clearance of the opacities four months after her bone marrow transplant (Figure 3). Her symptoms also subsided entirely. The patient's peripheral blood count showed complete chimerism.

## DISCUSSION

PAP due to familial MDS with GATA2 mutation is a rare disorder with the pathogenesis not fully understood. It is known that GATA2 belongs to the zinc finger transcription factors regulating macrophage phagocytosis. It is believed that the mutation in this gene can result in alveolar macrophage dysfunction. Therefore, a defective clearance and intra-alveolar accumulation of surfactant will ultimately accumulate of protein within the alveoli.^4^

The mean age of diagnosis is 51 years. Typically, 84% of the patients with S-PAP due to MDS will be symptomatic. The three most common symptoms are fever (45%), dyspnea on exertion (42%), and cough (42%).^5^ Physical examination may be normal; however, crackles (50%), clubbing (25%), and cyanosis (22%) may be found.^6^ Of those, the patient only presented with fever, dyspnea, cough, and her physical examination was normal.

As part of the initial investigation, the chest x-ray usually shows an alveolar filling pattern; however, interstitial, mixed diffuse nodular, and focal opacities are also described.^5^ HRCT is usually the following step. Diffuse ground-glass opacifications are usually found in patients with S-PAP, as seen in the case reported, while patchy geographic patterns are common in autoimmune PAP.^7^ The subpleural sparing and crazy-paving polygonal shapes with superimposed ground glass opacities delimited by thickened interlobular septa are more common in autoimmune PAP than in S-PAP,^8^ but were not seen in this patient's case. Bronchoalveolar lavage (BAL) should be performed if there is suspicion following HRCT, which classically reveals a milky-opaque return. Cytology may show foamy macrophages, acellular eosinophilic bodies, and PAS-positive debris.^9^^,^^10^ In a recent study involving 40 patients with both PAP and hematological malignancy, twenty-one were diagnosed with BAL, nine required transbronchial lung biopsy, and ten required video-assisted thoracoscopy.^11^ If BAL and cytology are nondiagnostic, a lung biopsy is the gold standard for diagnosis. As with our patient, biopsies show alveoli and terminal bronchioles filled with a granular and flocculent PAS-positive lipoproteinaceous material. Provided a typical history is associated with these observed imaging findings, biopsy results, negative anti-GM-CSF antibody, including identification of an underlying disease known to cause PAP, the diagnosis of S-PAP can be made.^3^^,^^9^ GM-CSF is a substance that mediates the surfactant clearance by macrophages. Patients with autoimmune PAP will present with antibodies against GM-CSF and, therefore impaired surfactant clearance. The negative anti-GM-CSF antibody is important to rule out autoimmune PAP.^3^

Treatment of S-PAP is focused on treating the cause.^3^^,^^9^ The use of whole lung lavage (WLL) can be used for symptomatic relief while treating the underlying cause.^5^^,^^12^ In the cases of GATA 2 deficient familial MDS and PAP, the earliest diagnosis and treatment with hematopoietic stem cell transplant can result in the release of functional macrophages and resolution of pulmonary alveolar proteinosis as seen in our patient.^13^^-^15

The long-term prognosis of S-PAP is uncertain, and seems to depend upon the etiology of the S-PAP. In a Japanese retrospective cohort of S-PAP due to MDS, 17 of 31 patients died, and their deaths were related to progression to acute myeloid leukemia (6 cases), PAP progression (6 cases), and pneumonia (11 cases) after a median follow-up of 40 months.^5^ Of the 14 surviving patients, only 7 had a bone marrow transplant. This study did not find any correlation between the MDS severity and the median survival, which was 13 months in patients with mild MDS and 15 months in severe MDS. The prognosis of S-PAP due to MDS is different from other non-secondary PAP. In a previous study^16^ comprising more than 300 patients with all causes of PAP, 7.9% of the cases had a spontaneous resolution, and about 80% of patients who received lavage attained significant improvement. The overall survival rates at 2, 5, and 10 years were 78.9%, 74.7%, and 68.3%.

## CONCLUSION

S-PAP diagnosis should be considered in patients with myelodysplastic syndrome presenting with dyspnea and ground-glass opacities on lung CT scans. PAP often provides a nidus for infection, increasing morbidity. S-PAP carries a worse prognosis than primary PAP.^5^ However, a previous cohort^5^ show favorable outcomes if these patients are early diagnosed and treated in a good general clinical condition. Our case report highlights that PAP can be reversed with timely management of the underlying myelodysplastic syndrome, inferring recovery of alveolar macrophages following pluripotent stem cell transplantation.
